# NT5DC2 promotes tumor cell proliferation by stabilizing EGFR in hepatocellular carcinoma

**DOI:** 10.1038/s41419-020-2549-2

**Published:** 2020-05-07

**Authors:** Kang-Shuai Li, Xiao-Dong Zhu, Hong-Da Liu, Shi-Zhe Zhang, Xiao-Long Li, Nan Xiao, Xue-Feng Liu, Bin Xu, Ming Lei, Yuan-Yuan Zhang, Wen-Kai Shi, Man-Qing Cao, Yun-Fei Xu, Zhao-You Tang, Hui-Chuan Sun

**Affiliations:** 1 Department of Liver Surgery and Transplantation, Liver Cancer Institute, Zhongshan Hospital, Fudan University, 200032 Shanghai, China; 2 Key Laboratory of Carcinogenesis and Cancer Invasion (Fudan University), Ministry of Education, 200032 Shanghai, China; 3 Department of General Surgery, The First Affiliated Hospital of Nanjing Medical University, 210029 Nanjing, Jiangsu province China; 4 Department of General Surgery, Qilu Hospital of Shandong University, No. 107, Wenhua Xi Road, 250012 Jinan, China

**Keywords:** Liver cancer, Liver cancer

## Abstract

Most hepatocellular carcinoma (HCC) patients are diagnosed at an advanced stage; however, the effect of systemic therapy on advanced HCC remains undetermined. Therefore, new treatment targets must be identified. We analyzed Gene Expression Omnibus datasets from two HCC patient cohorts and found that NT5DC2 was associated with vascular invasion and poor survival. In two hepatoma cell lines, NT5DC2 overexpression promoted HCC cell proliferation and clone formation in vitro and promoted tumor growth in vivo. Coimmunoprecipitation assays and liquid chromatography with tandem mass spectrometry analysis revealed that NT5DC2 bound directly to epidermal growth factor receptor (EGFR). NT5DC2 upregulated EGFR expression by downregulating EGFR ubiquitination and preventing its degradation via the ubiquitin-proteasome pathway but did not upregulate its transcription. EGFR upregulation activated downstream signal transduction, which played a critical role in the protumor effects of NT5DC2. Erlotinib, a small-molecule inhibitor of EGFR, blocked the effect of NT5DC2 in promoting HCC cell proliferation. In a cohort of 79 patients who underwent curative resection for HCC, NT5DC2 expression in the tumors was associated with larger tumors and microvascular invasion. NT5DC2 expression was also independently associated with recurrence-free survival. The present study demonstrated for the first time that NT5DC2 promotes tumor cell proliferation in HCC and may serve as a potential molecular target for treating HCC. EGFR blockage could be used to treat selected patients with NT5DC2 upregulation.

## Introduction

Liver cancer ranks sixth in incidence and fourth in mortality globally among all malignant tumors^1^. According to the Global Burden of Disease Cancer Collaboration, liver cancer was the second leading cause of absolute years of life lost among both sexes in 2017^2^. Hepatocellular carcinoma (HCC) is the primary pathological type of liver cancer. The treatment regimen for hepatocellular carcinoma includes surgery, liver transplantation, interventional therapy, radiotherapy and systemic supportive therapy^3^. Although surgical resection ranks as the most effective treatment, it benefits only some HCC patients because most patients are diagnosed at advanced stages and have lost the window for surgical treatment and thus must receive systemic therapy^4^. Molecular targeted therapy is a promising area that has shown progress in treating malignant tumors^5,6^. However, few molecular targeted drugs have been approved by the Food and Drug Administration to treat HCC, and these drugs mainly include tyrosine kinase inhibitors^7–9^. The median survival time of patients who receive systemic therapy is less than 16 months; therefore, identifying additional potential treatment targets will expand the treatment selection and provide insight into the HCC carcinogenesis^4^.

The epidermal growth factor receptor (EGFR; also known as ErbB1 or HER1) is a member of the EGFR/ErbB subfamily of receptor tyrosine kinases (RTKs)^10^. EGFR is a receptor for EGF, transforming growth factor-α, and amphiregulin^11^. EGFR activation induces homodimerization or heterodimerization with other related RTKs and leads to EGFR phosphorylation at multiple tyrosine residues in its intracellular region^12^. Subsequently, signaling molecules including GRB2, PI3K, and PLC are recruited, and multiple downstream signaling pathways, including the Ras-ERK and PI3K-AKT pathways, are initiated, which promote cell proliferation, survival, migration and other cellular processes^11,13^. EGFR malfunction plays an essential role in the development of various cancers^14^. Small-molecule tyrosine kinase inhibitors targeting EGFR have been developed and successfully applied to treat lung cancer, colorectal cancer, and pancreatic cancer^15^. However, although several preclinical experimental studies revealed the treatment potential of EGFR inhibitors for HCC, in clinical trials, patients with advanced HCC received no treatment benefits from EGFR inhibitors. Thus, a better understanding of EGFR signaling regulation in HCC is needed^16^.

5’-Nucleotidase domain containing 2 (NT5DC2) is a member of the NT5DC family and contains a haloacid dehalogenase motif localized in the N-terminus of these proteins^17^. NT5DC2 is associated with attention-deficit/hyperactivity disorder and bipolar disorder^18,19^. Recently, a study showed that NT5DC2 interacts with tyrosine hydroxylase (TH) to regulate TH catalytic activity and thus regulate catecholamine synthesis^20^. NT5DC2 has been shown to interact with and stabilize Fyn, a Src family proto-oncogene, and plays a role in regulating glioblastoma progression^21^. However, to date, little is known about the role of NT5DC2 in HCC development and progression.

In this study, two Gene Expression Omnibus (GEO) datasets were analyzed for targets associated with both vascular invasion and poor prognosis in HCC, and NT5DC2 was identified as a promising treatment candidate. Overexpression of NT5DC2 promotes HCC proliferation by altering the cell cycle, while downregulation of NT5DC2 reverses this process. In vivo studies have shown that overexpression of NT5DC2 promotes tumor growth, and downregulation of NT5DC2 inhibits tumor growth. The major underlying mechanism is that NT5DC2 binds to EGFR and upregulates EGFR expression by downregulating EGFR ubiquitination. NT5DC2 also promotes EGFR downstream signal transduction and increases the phosphorylation levels of ERK and Akt. Importantly, upregulation of NT5DC2 in tumor tissue indicates a poor prognosis.

## Results

### NT5DC2 was associated with HCC progression

**Fig. 1:**
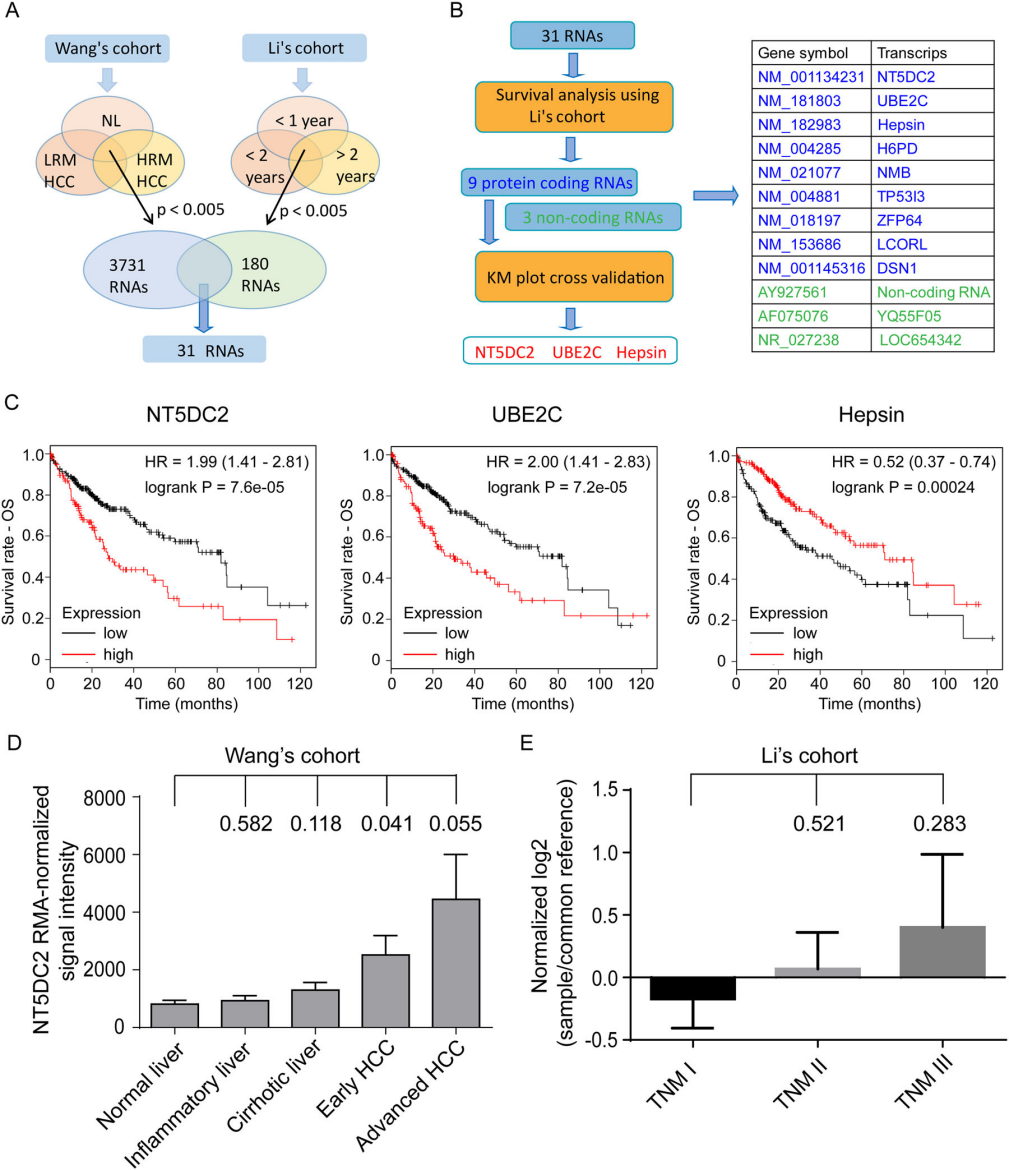
Identification of NT5DC2 as a potential target for treating HCC. **a** Schematic graph showing the identification of differentially expressed RNAs after analyzing two GEO databases (GSE54238 for Wang’s cohort, GSE40144 for Li’s cohort). **b** Flowchart showing the identification of NT5DC2, UBE2C and hepsin as candidate genes. Twelve genes were found significantly associated with OS and DFS in Li’s cohort. Further cross-validation of patient OS using K–M plot identified NT5DC2, UBE2C and hepsin as candidate genes. **c** Candidate genes significantly associated with patient’s OS in K–M plot database (http://www.kmplot.com/). **d** NT5DC2 expression in normal liver, inflammatory liver, liver with cirrhosis, early HCC and advanced HCC in Wang’s cohort. Student’s *t*-tests were performed to analyze the differences between each group and normal liver. *P* values are labeled above the column for each group. **e** NT5DC2 expression in TNM stage I patients, stage II patients and stage III patients in Li’s cohort. Student’s *t*-tests were performed to analyze the differences between each group and TNM stage I patients. *P* values are labeled above the column for each group.

Venous metastasis is an important tumor feature associated with early tumor recurrence and poor prognosis after resection^22^. To identify genes associated with early recurrence and novel potential targets of HCC, two GEO datasets, Wang’s cohort (GSE54238)^23,24^, which contains gene expression data for HCC with or without venous metastasis, and Li’s cohort (GSE40144), which contains recurrence and survival data, were analyzed using GEO2R online software. The expression profiles of normal liver tissue, low-risk metastatic (LRM) HCC, which refers to primary HCC without venous metastases and no recurrence within 2 years after liver resection, and high-risk metastatic (HRM) HCC, which refers to primary HCC with macroscopic tumor thrombus in the major branch of the portal vein or inferior vena cava at diagnosis, were compared to identify genes associated with early recurrence in the GSE54238 dataset^24^. The expression profiles of tumors from patients with a DFS of less than 1 year, more than 1 year and less than 2 years, and more than 2 years in the GSE40144 dataset were also compared to identify genes associated with early recurrence. In total, 3731 differentially expressed genes with *p* < 0.005 were identified in dataset GSE54238, and 180 differentially expressed genes with *p* < 0.005 were identified in dataset GSE40144. Thirty-one genes were common in the differential expression profiles for both datasets (Fig. 1a).

The correlation between gene expression and patient OS and DFS were analyzed in the GSE40144 dataset. Nine protein-coding genes and three noncoding genes were associated with patient OS and DFS (Fig. 1b and Supplemental Fig. 1). The correlation between nine protein-coding genes and patient survival was further cross-validated through the K–M plotter which consists of HCC cohort from TCGA database^25,26^ (http://www.kmplot.com/), and NT5DC2, UBE2C and hepsin were identified as candidate genes for further study (Fig. 1b, c). NT5DC2 is a functionally unknown gene, while the roles of UBE2C and hepsin have been extensively studied^27–29^; thus, NT5DC2 was chosen for further study.

Analyzing the GSE54238 dataset showed that the NT5DC2 expression levels were elevated in the tumors compared with those in the nontumor tissues (Fig. 1d). Analyzing the GSE40144 dataset showed that NT5DC2 expression was associated with tumor node metastasis (TNM) stages although the difference didn’t reach a significant level (Fig. 1e). The correlation between the expression of NT5DC2 and oncogenic markers including DANCR, AHCYL2, LAMP2, SPRY1, SERPINA7, FGGY, and ASLNC16648 which were typically tested for Wang’s cohort were also analyzed. The results showed that NT5DC2 expression correlates with AHCYL2 expression (Supplemental Fig. 2).

### Upregulation of NT5DC2 facilitated HCC cell proliferation in vitro and HCC cell growth in vivo

**Fig. 2:**
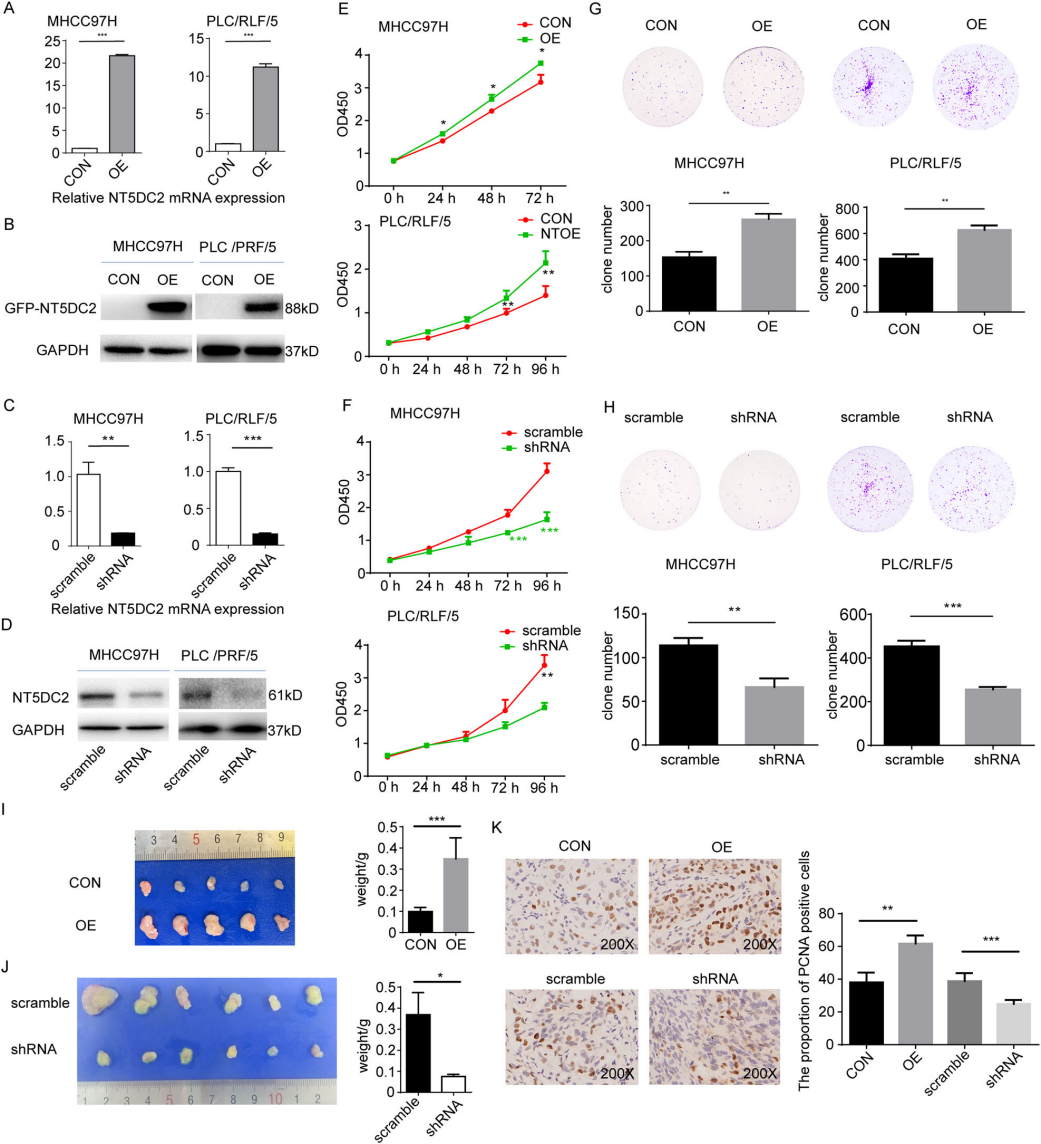
Upregulation of NT5DC2 facilitated HCC cell proliferation in vitro and HCC cell growth in vivo, while NT5DC2 knockdown reversed this process. **a, b** MHCC97H and PLC/RLF/5 cell lines were overexpressed with GFP (CON) or GFP-tagged NT5DC2 (OE). Verification of NT5DC2 overexpression (OE) in MHCC97H and PLC/RLF/5 cell lines at both the mRNA (**a**) and protein levels (**b**). **c**, **d** Verification of NT5DC2 knockdown (shRNA) in MHCC97H and PLC/RLF/5 cell lines at both the mRNA (**c**) and protein levels (**d**). **e** CCK8 assays for cell proliferation of MHCC97H-NT5DC2-overexpression and PLC/RLF/5-NT5DC2-overexpression cells compared with their vector controls (CON). NT5DC2 promoted liver cancer cell proliferation in vitro. **f** CCK8 assays for cell proliferation of MHCC97H-NT5DC2-knockdown and PLC/RLF/5-NT5DC2-knockdown cells compared with their vector controls. NT5DC2 knockdown inhibited liver cancer cell proliferation in vitro. **g** Clone formation of MHCC97H-NT5DC2-overexpression and PLC/RLF/5-NT5DC2-overexpression cells compared with their vector controls. NT5DC2 promoted cell clone formation in vitro. **h** Clone formation of MHCC97H-NT5DC2-knockdown and PLC/RLF/5-NT5DC2-knockdown cells compared with their vector controls. NT5DC2 knockdown inhibited cell clone formation in vitro. **i** MHCC97H cells with NT5DC2 overexpression (OE) or its vector control (CON) were subcutaneously implanted into nude mice, and the tumors were harvested 3 weeks later (left). Compared with the controls, NT5DC2 overexpression significantly promoted tumor growth (right). **j** MHCC97H cells with NT5DC2 knockdown (shRNA) or its vector control (scramble) were subcutaneously implanted into nude mice, and the tumors were harvested 5 weeks later (left). Compared with the controls, NT5DC2 knockdown significantly inhibited tumor growth (right). **k** Tumor slides were analyzed via immunohistochemistry for PCNA expression (left). Compared with the controls, NT5DC2 overexpression significantly increased the proportions of PCNA-positive cells, while NT5DC2 knockdown significantly decreased the proportions of PCNA-positive cells (right). *p < 0.05; **p < 0.01; ***p < 0.001.

Because NT5DC2 expression was associated with patient survival, we further investigated the mechanism by which NT5DC2 regulates HCC progression. First, NT5DC2 expression on the normal cell line L02 and hepatoma cell lines including MHCC97H and PLC/RLF/5 were examined by Western blot. The results identified a substantial upregulation of NT5DC2 in the hepatoma cell lines than normal cell line (Supplemental Fig. 3). NT5DC2-overexpression and NT5DC2-knockdown cell lines were established in MHCC97H and PLC/RLF/5 cell lines. The overexpression and knockdown efficiencies were validated via qPCR (Fig. 2a–c) and western blot (Fig. 2b, d). A CCK8 assay was performed to evaluate the effect of NT5DC2 overexpression on HCC cell proliferation (Fig. 2e). NT5DC2 promoted the proliferation of both MHCC97H and PLC/RLF/5 cell lines in vitro. Downregulation of NT5DC2 decreased cell proliferation in both cell lines (Fig. 2f). Clone formation assays showed that the clone formation ability was increased in NT5DC2-overexpressing MHCC97H and PLC/RLF/5 cells compared with that of the control cells (Fig. 2g). The clone formation ability of the NT5DC2-knockdown cells was significantly weakened compared with that of the scramble cells (Fig. 2h).

Subcutaneous xenograft tumor models were established to explore the effects of NT5DC2 on HCC progression in vivo. After three weeks (for knockdown and control cells) or 5 weeks (for overexpression and control cells) of tumor incubation, the mice were sacrificed. Tumor weights in the MHCC97H-OE group were significantly larger than those in the MHCC97H-control group, and the tumor weights in the MHCC97H-shRNA group were significantly less than those of the MHCC97H-scramble group (Fig. 2i, j). Immunohistochemical staining revealed that PCNA expression was markedly increased in MHCC97H cells transfected with NT5DC2 and decreased in the NT5DC2 knockdown cells (Fig. 2k), indicating that NT5DC2 promoted HCC growth in vivo.

**Fig. 3:**
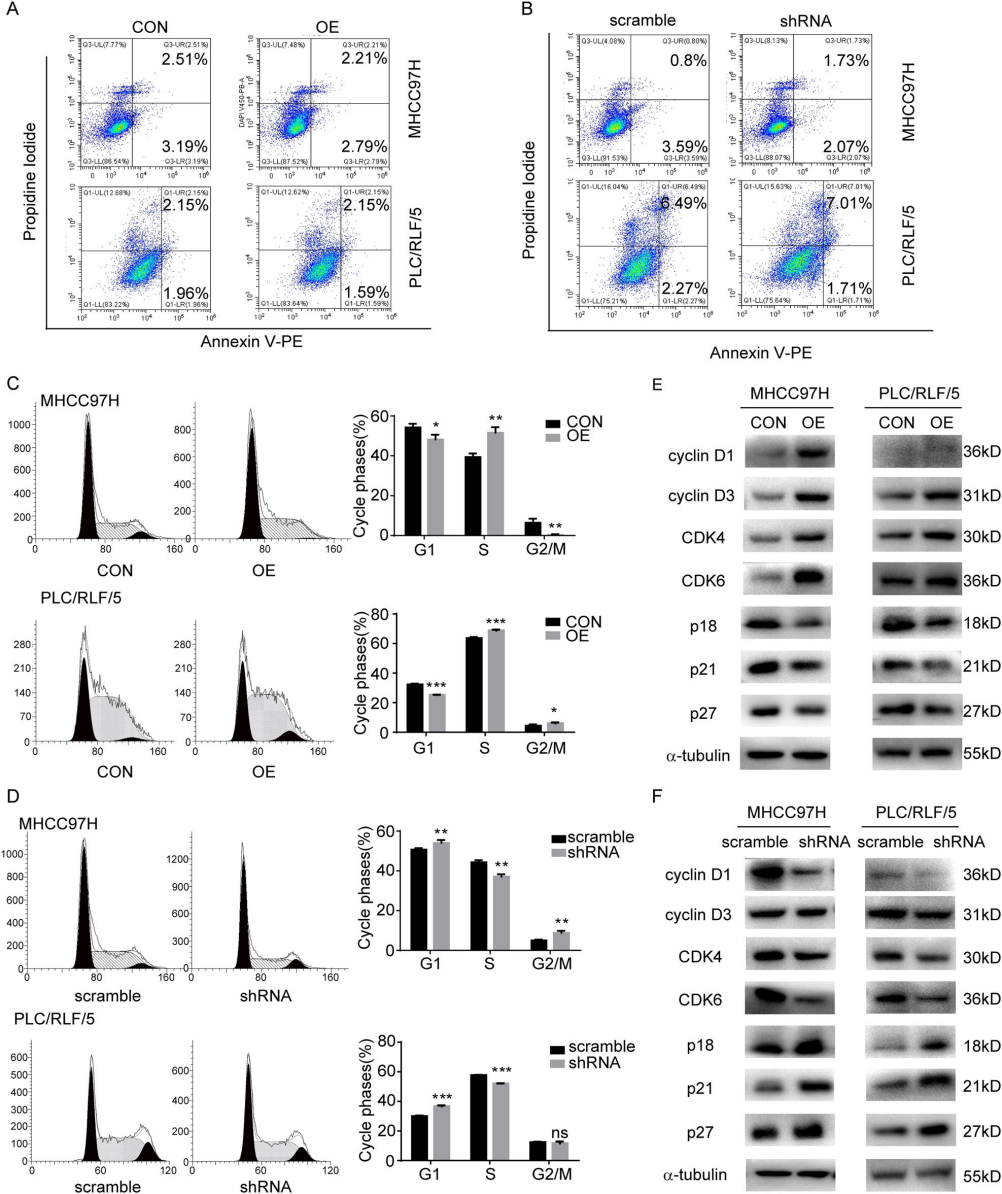
NT5DC2 upregulation did not affect apoptosis but facilitated cell cycle transition from the G1 to S phase in HCC cells; NT5DC2 knockdown reversed this process. **a** Flow cytometry for detecting apoptotic rates in MHCC97H-NT5DC2-overexpressing and PLC/RLF/5-NT5DC2-overexpressing cells compared with their vector controls. NT5DC2 did not affect the apoptotic rates in these two cell lines. **b** Flow cytometry for detecting apoptotic rates in MHCC97H-NT5DC2-knockdown and PLC/RLF/5-NT5DC2-knockdown cells compared with their vector controls. NT5DC2 knockdown did not affect the apoptotic rates in liver cancer cells. **c** Flow cytometry for detecting cell cycle ratios in MHCC97H-NT5DC2-overexpressing and PLC/RLF/5-NT5DC2-overexpressing cells compared with their vector controls (left). Statistical analysis of cell cycle phase ratios in MHCC97H-NT5DC2-overexpressing and PLC/RLF/5-NT5DC2-overexpressing cells (right). *p < 0.05; **p < 0.01; ***p < 0.001. **d** Flow cytometry for detecting cell cycle ratios of MHCC97H-NT5DC2-knockdown and PLC/RLF/5-NT5DC2-knockdown cells compared with their vector controls (left). Statistical analysis of cell cycle phase ratios in MHCC97H-NT5DC2-knockdown and PLC/RLF/5-NT5DC2-knockdown cells (right). *p < 0.05; **p < 0.01; ***p < 0.001. **e** Western blot analysis of the effects of NT5DC2 overexpression on the expressions of cell cycle-regulating proteins cyclin D1, cyclinD3, CDK4, CDK6, p18, p21, and p27. **f** Western blot analysis of the effects of NT5DC2 knockdown on the expressions of cell cycle-regulating proteins cyclin D1, cyclinD3, CDK4, CDK6, p18, p21, and p27.

To further explore the underlying mechanism by which NT5DC2 affects cell proliferation, apoptosis and the cell cycle were analyzed. The apoptotic rate did not differ between the cells overexpressing NT5DC2 and the vector control cells in both the MHCC97H and PLC/RLF/5 cell lines (Fig. 3a). The NT5DC2-knockdown cells and vector control cells also showed no difference (Fig. 3b). We also examined cell death in the subcutaneous tumor sections through TUNEL staining. As shown in supplemental Fig. 4, no difference in cell death was observed between control and overexpression group. There is also no difference between scramble and shRNA group. Whether NT5DC2 promoted HCC cell proliferation by altering the cell cycle phase was further analyzed via flow cytometry. NT5DC2 overexpression decreased the G1 phase ratio and increased the S phase ratio in both the MHCC97H and PLC/RLF/5 cell lines, indicating that NT5DC2 promoted HCC cell proliferation by releasing the cells from the G0/G1 phase block (Fig. 3c and Supplemental Fig. 5). NT5DC2 knockdown increased the G1 phase ratio and decreased the S phase ratio in both cell lines, further confirming the results for the overexpression (Fig. 3d). The cell cycle was regulated by a series of regulatory proteins, including cell cycle-promoting proteins cyclin D1, cyclin D3, CDK4, and CDK6 and cell cycle-arresting proteins p18, p21 and p27. Western blot analysis was then performed to examine the expression of these proteins in both NT5DC2-overexpressing and NT5DC2-knockdown cells. NT5DC2 overexpression promoted the expression of cell cycle-regulating proteins (Fig. 3e), while NT5DC2 knockdown inhibited the expression of cell cycle-regulating proteins (Fig. 3f). These results indicate that NT5DC2 upregulation promotes HCC tumor cell proliferation in vitro.

### NT5DC2 promoted HCC proliferation by upregulating EGFR expression

**Fig. 4:**
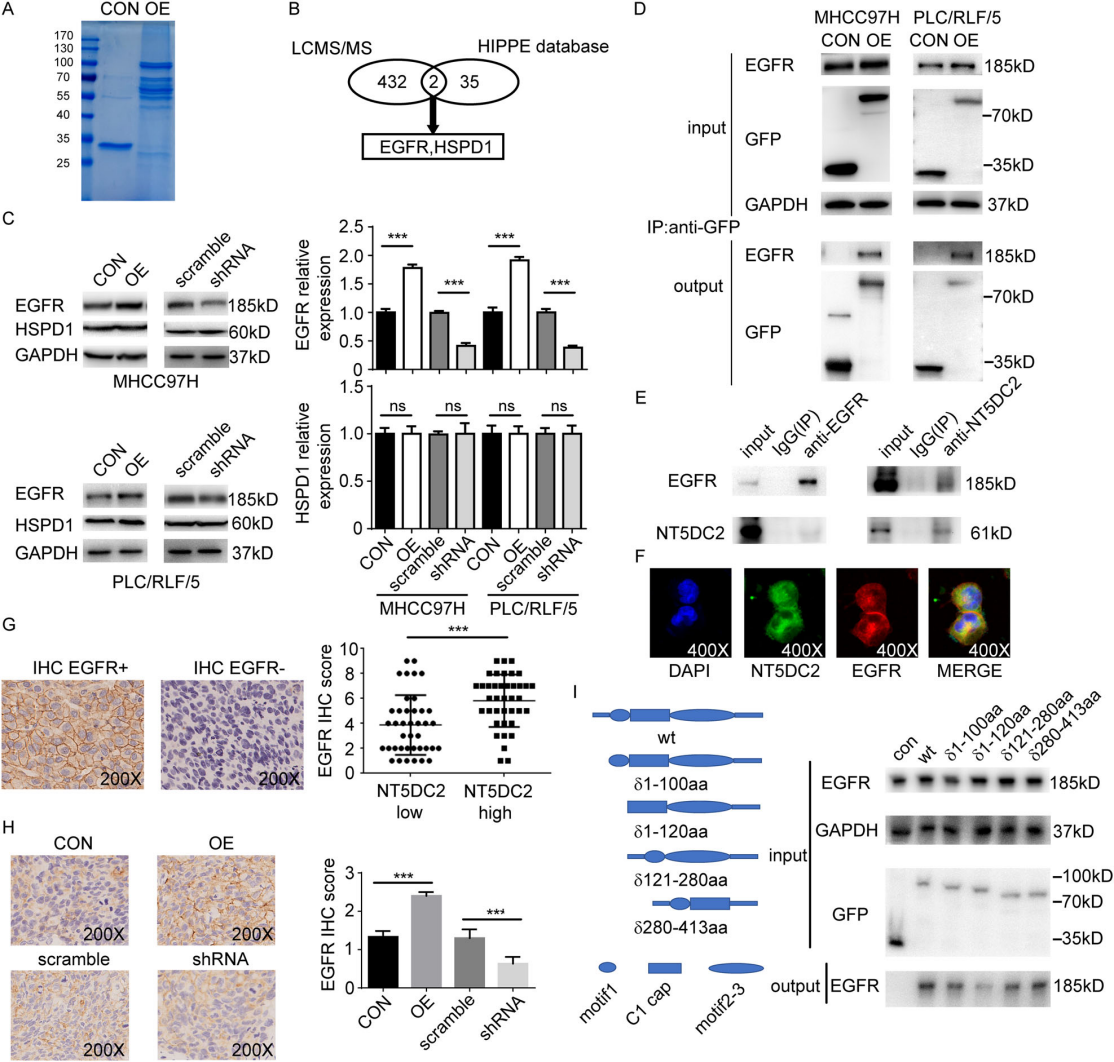
NT5DC2 binds to EGFR and regulates EGFR expression. **a** Coomassie blue staining of anti-GFP coimmunoprecipitation in MHCC97H cells overexpressing GFP-NT5DC2 or a GFP vector control. **b** Schematic graph showing the identification of EGFR and HSPD1 as potential NT5DC2-reacting proteins via LC-MS/MS and the HIPPIE database. **c** Western blot analysis of EGFR and HSPD1 expression in NT5DC2-overexpressing and NT5DC2-knockdown cells in both MHCC97H and PLC/RLF/5 cell lines (left). EGFR had higher expression in NT5DC2-overexpressing cell lines and lower expression in NT5DC2-knockdown cell lines (right, upper), while HSPD1 expression was unchanged in both NT5DC2-overexpressing and NT5DC2-knockdown cell lines (right, lower). **d** Coimmunoprecipitation assays were performed in MHCC97H and PLC/RLF/5 cells transfected with a vector containing GFP-tagged NT5DC2 or an empty vector. **e** Co-immunoprecipitation assays were performed in PLC/RLF/5 cells, IgG was used as a control. Endogenous EGFR was co-immunoprecipitated by the anti-NT5DC2, while the endogenous NT5DC2 was reciprocally co-immunoprecipitated by the anti-EGFR antibody. **f** Co-localization of NT5DC2 (green) with EGFR (red) in PLC/RLF/5 cells on confocal microscopy. **g** NT5DC2 expression was determined via qPCR, while EGFR expression was determined via IHC (upper) in 79 human tumor tissues. EGFR expression was higher in the NT5DC2-high group (lower). **h** Slide sections were analyzed via IHC for EGFR expression in subcutaneous MHCC97H tumors (left). NT5DC2-overexpressing tumors had relatively higher EGFR scores, while NT5DC2 knockdown tumors had relatively lower EGFR scores on the IHC (right; magnification ×200). **i** Control or different NT5DC2 truncations with GFP tag were transfected into PLC/RLF/5 cells. Co-immunoprecipitation assays were performed with anti-GFP beads.

To explore the molecular mechanism by which NT5DC2 contributes to HCC growth, immunoprecipitation and LC-MS/MS analyses were performed on HCC cells with stably overexpressed NT5DC2 or a vector control to identify compositional differences in the protein complexes (Fig. 4a). NT5DC2-associated complexes were isolated from MHCC97H cells using agarose beads coated with anti-GFP monoclonal antibodies (mAbs). The mAb-coated beads recruited NT5DC2 and associated proteins in live cells, and the complexes were then extracted, purified and analyzed via MS.

Proteomic analysis identified 457 proteins with Mascot scores over 60 in complexes associated with NT5DC2 overexpression and 31 in complexes associated with the vector control. Overlapped proteins (23 proteins; 5%) were identified in complexes associated with NT5DC2 overexpression and their vector controls; thus, 434 proteins that were uniquely bound to NT5DC2 were identified. Human Integrated Protein-Protein Interaction rEference (HIPPIE) is a web tool that generates reliable protein-protein interactions based on experimental data. To further identify unique and authentic proteins combined with NT5DC2, the HIPPIE database was searched for proteins that interacted with NT5DC2, and 37 proteins were identified. The HIPPIE data revealed two overlapped proteins: EGFR and HSPD1. Thus, EGFR and HSPD1 were used for further study (Fig. 4b).

EGFR and HSPD1 expression in the NT5DC2-overexpressing and NT5DC2-knockdown cells in both the MHCC97H and PLC/RLF/5 cell lines were examined. NT5DC2 overexpression increased EGFR expression, and NT5DC2 knockdown reduced EGFR expression, while neither overexpression nor knockdown of NT5DC2 altered the HSPD1 expression (Fig. 4c). Therefore, we hypothesized that NT5DC2 promotes tumor growth by physically associating with EGFR and facilitating EGFR expression.

To test this hypothesis, we further validated the results via repeated immunoprecipitation followed by western blotting of HCC cells. Endogenous EGFR was coimmunoprecipitated using the anti-GFP antibody in NT5DC2-overexpressing MHCC97H and PLC/RLF/5 cells (Fig. 4d). To test whether endogenous NT5DC2 binds to EGFR, we further performed coimmunoprecipitation assay. Endogenous EGFR were co-immunoprecipitated by the anti-NT5DC2 antibody, whereas the endogenous NT5DC2 was reciprocally co-immunoprecipitated by the anti-EGFR antibodies in PLC/RLF/5 cells (Fig. 4e), respectively. Confocal microscopy demonstrated NT5DC2 localization at the cell membrane and in the cytoplasm and colocalization of NT5DC2 with EGFR in HCC cells was observed (Fig. 4f). The correlation between NT5DC2 expression and EGFR expression in 79 HCC tumor tissues was examined. NT5DC2 expression was determined via qPCR, and EGFR expression was determined via immunohistochemistry (IHC). Patients with high NT5DC2 expression had higher EGFR expression (Fig. 4g). Furthermore, tumor tissue with NT5DC2 overexpression collected from subcutaneous xenograft tumor models had significantly higher EGFR expression, while tumor tissue with NT5DC2 knockdown had significantly lower EGFR expression compared with that of their vector controls (Fig. 4h). NT5DC2 belongs to 5’-nucleotidase family and shows sequence similarity with NT5C2 which is a protein well studied. The core catalytic region of NT5C2 consists of 4 motifs including motif 1, C1 cap, motif 2 and motif 3^30^. To further identify the interacting domain of NT5DC2 with EGFR, a sequence comparison of NT5DC1, NT5DC2, NT5DC3, NT5DC4, and NT5C2 was performed (Supplemental Fig. 6). Meanwhile, by referring to the published crystal structure of NT5C2 (pdb code: 2j2c), a series of truncation constructs omitting motif 1, C1 cap and motif 2–3 separately was designed (Fig. 4i). These constructs were then introduced into PLC/RLF/5 cell line and immunoprecipitation assay was performed. Although, all 3 constructs affect the combination of NT5DC2 with EGFR, motif 1was the most significant one (Fig. 4i). Taken in all, these data demonstrated that NT5DC2 physically associates with EGFR and modulates its expression.

### NT5DC2 upregulated EGFR expression by downregulating EGFR ubiquitination

**Fig. 5:**
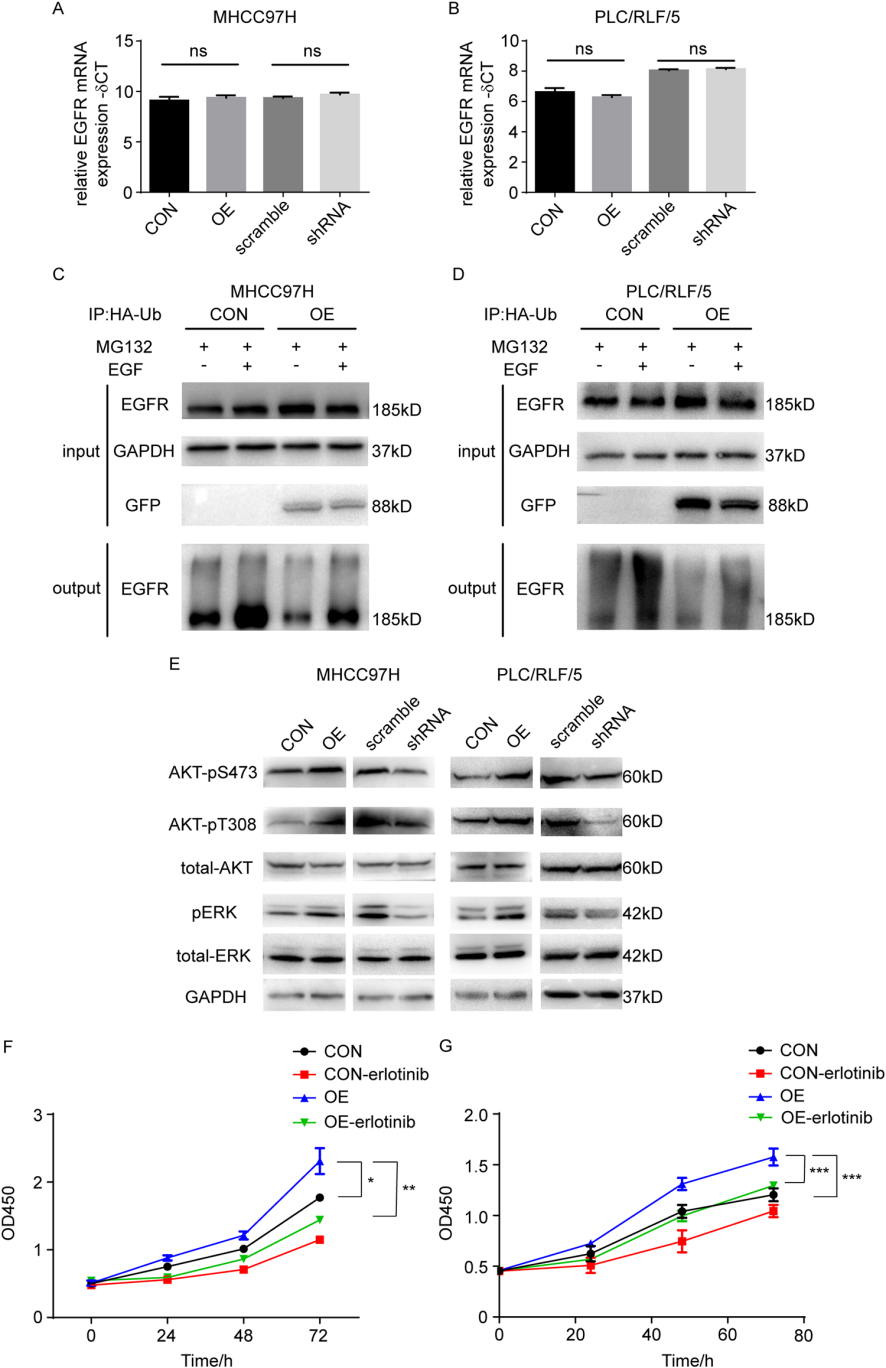
NT5DC2 inhibited EGFR ubiquitin and promoted EGFR downstream signaling, while erlotinib blocked the effect of NT5DC2 on cell proliferation. **a**, **b** qPCR analysis of EGFR mRNA levels in NT5DC2-overexpression and NT5DC2-knockdown cells in both MHCC97H (**a**) and PLC/RLF/5 (**b**) cell lines. **c**, **d** Western blot analysis of EGFR protein ubiquitination levels in MHCC97H cells (**c**) and PLC/RLF/5 cells treated with MG132 (10 μmol/L, 8 h) with or without EGF (25 ng/ml, 8 h) with the indicated antibodies. **e** Phosphorylation levels of EGFR signaling pathway-associated proteins, including AKT-pS473, AKT-pT308, and pERK, were detected via western blot in NT5DC2-overexpressing and NT5DC2-knockdown cells compared with their vector controls in both MHCC97H and PLC/RLF/5 cell lines. **f**, **g** CCK8 assays were performed to characterize the inhibitory effects of erlotinib on the proliferation of NT5DC2-overexpressing MHCC97H (**f**) and PLC/RLF/5 (**g**) cells.

To further identify the underlying mechanism of regulating EGFR expression, we determined whether NT5DC2 upregulates EGFR by promoting EGFR transcription. Quantitative PCR was performed in MHCC97H and PLC/RLF/5 cell lines with NT5DC2 overexpression or knockdown. The data revealed no changes in the EGFR mRNA levels (Fig. 5a, b). Ubiquitination-mediated degradation of EGFR is a main regulating mechanism of EGFR expression^31,32^. Thus, we hypothesized that NT5DC2 upregulates EGFR expression by downregulating the EGFR ubiquitin. HA-tagged ubiquitin was overexpressed in MHCC97H and PLC/RLF/5 cell lines overexpressing NT5DC2 and immunoprecipitated with anti-HA antibody-coated magnetic beads. Western blot analysis revealed that EGFR was significantly ubiquitinated after stimulating EGF (Fig. 5c, d), whereas the ubiquitination level of EGFR was significantly decreased in both MHCC97H and PLC/RLF/5 cell lines overexpressing NT5DC2 (Fig. 5c, d). These results revealed that NT5DC2 upregulated EGFR expression by downregulating EGFR ubiquitin.

### NT5DC2 overexpression promoted downstream signal transduction of EGFR, and erlotinib blocked the effect of NT5DC2 on cell proliferation

We measured the relative phosphorylation of the EGFR downstream target proteins, AKT and ERK, to further validate the above data. NT5DC2 overexpression significantly increased AKT and ERK phosphorylation (Fig. 5e), indicating that NT5DC2 regulates activation of the EGFR signaling pathways. Control and NT5DC2 overexpressing MHCC97H and PLC/RLF/5 cells were then stimulated with 25 ng/ml EGF and CCK8 assay was performed. As shown in the supplemental Fig. 7, EGF stimulation promoted the cell proliferation in both control and NT5DC2 overexpressing cells while EGF stimulation showed more promoting effect on NT5DC2 overexpressing cells. Erlotinib is a small-molecule EGFR tyrosine kinase inhibitor that inhibits cancer cell growth and metastasis. To determine whether the proliferative effects of NT5DC2 on HCC cells were EGFR-dependent, MHCC97H-NT5DC2 cells, PLC/RLF/5-NT5DC2 cells and their control cells were treated with erlotinib (30 μM). CCK8 assays demonstrated that erlotinib reduced the promotive effect of NT5DC2 on cell proliferation (Fig. 5f, g). These results also demonstrated that NT5DC2 promoted HCC cell proliferation through EGFR-mediated signaling pathways. These results indicated that NT5DC2 activated EGFR-mediated signaling pathways by regulating EGFR stability by reducing EGFR ubiquitination levels.

### High NT5DC2 expression was associated with poor patient survival

**Fig. 6:**
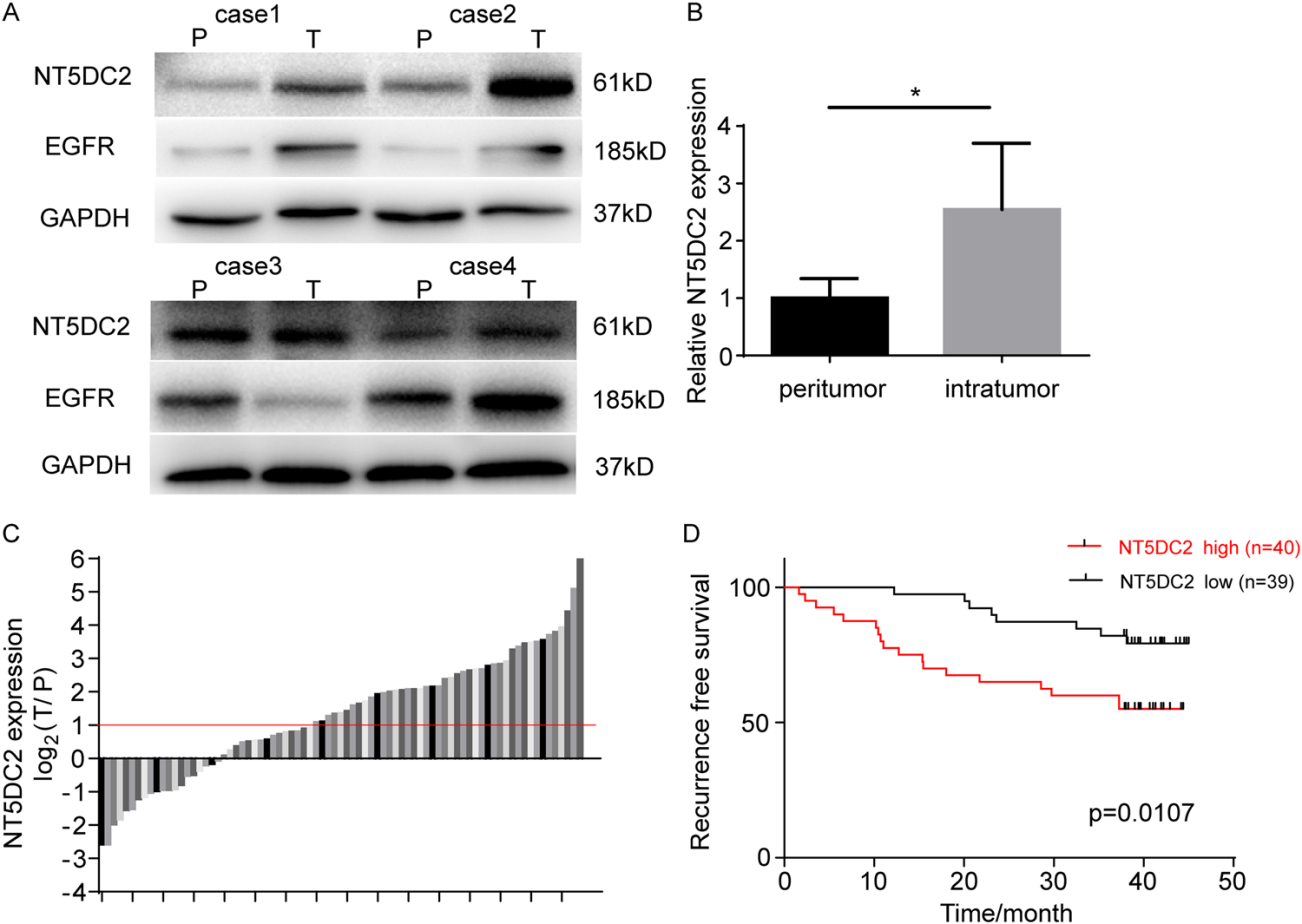
NT5DC2 expression was associated with poor patient survival. **a**, **b** Western blot (**a**) and statistical analysis of NT5DC2 and EGFR expression in four paired peritumoral (P) and tumor (T) tissues. **c** qPCR analysis of NT5DC2 mRNA expression levels in 79 paired surgically removed tumor and peritumoral liver tissues. **d** The relationship between NT5DC2 expression and RFS after surgery.

NT5DC2 expression in 4 paired tumor and peritumoral tissues were analyzed at the protein level via western blot. Tumoral tissues showed significantly higher NT5DC2 expression compared with that of peritumoral tissues (Fig. 6a, b). EGFR expression was also upregulated in 3 of the 4 cases (Fig. 6a). The mRNA expression level of NT5DC2 was also compared in 79 paired HCC tissues and adjacent nontumor liver tissues. The mean NT5DC2 mRNA levels in 44 HCC cases were significantly higher (more than two folds; i.e., log2 [fold change] > 1) than those in the adjacent nontumor liver tissues (55.7%; Fig. 6c).

To illustrate the clinical relevance of NT5DC2 expression in HCC, patients were separated into high and low expression groups based on NT5DC2 mRNA expression levels by median expression. Associations between NT5DC2 expression and clinicopathologic characteristics of HCC were evaluated. Patients with higher NT5DC2 expressions had larger tumors and higher incidences of microvascular invasion (Table 1). Survival analysis indicated that patients with higher NT5DC2 expression in HCC tissues had shorter DFS times than did those with low expression (Fig. 6d). Multivariate analysis identified NT5DC2 expression as an independent risk factor for DFS (HR = 2.605, 95% CI, 1.124–6.040, *P* = 0.026). Thus, high NT5DC2 expression was associated with invasive characteristics and a poor prognosis in HCC patients. Because the median follow-up time for this cohort was 41.3 months, the OS data were insufficient, and the association between NT5DC2 expression and OS was not analyzed.

**Table 1:** Correlation between NT5DC2 expression in tumor cells and clinicopathological features in HCC patients.

Parameters	NT5DC2 expression		*P* value
	High	Low	
Sex			
Male	31	31	0.830
Female	9	8	
Age (years)			
<50	9	8	0.830
≥50	31	31	
Cirrhosis			
Absent	6	6	0.962
Present	34	33	
Tumor number			
Single	38	35	0.325
Multiple	2	4	
Tumor size			
<3 cm	16	25	0.032*
≥3 cm	24	14	
Microvascular invasion			
Absent	25	33	0.026*
Present	15	6	
HBsAg			
Negative	7	6	0.800
Positive	33	33	
AFP			
<20 ng/ml	16	20	0.314
≥20 ng/ml	24	19	

*HBsAg* hepatitis B surface antigen, *AFP* alpha fetoprotein.

## Discussion

Venous metastasis and early recurrence are associated with poor outcomes for HCC patients^22,24^. Therefore, genes associated with both venous metastasis and early recurrence may be valuable treatment targets and prognostic factors^24^. In this study, the expression profiles of lncRNAs/mRNAs were analyzed for genes that were differentially expressed between normal liver tissue, tumor tissue without microvascular invasion, and tumor tissue with macrovascular invasion to identify genes associated with venous metastasis. Recurrence within 2 years after liver tumor resection is believed to be associated with primary tumors, whereas recurrence after 2 years is believed to indicate new tumor lesions^33,34^. In another cohort, expression profiles of lncRNAs/mRNAs were analyzed for differentially expressed genes among patients whose DFSs were less than 1 year, more than 1 year and less than 2 years, or more than 2 years to identify genes associated with early recurrence. Common genes associated with both venous metastasis and early recurrence were identified and validated with independent databases, including GEO, the European Genome-phenome Archive, and the Cancer Genome Atlas.

NT5DC2 belongs to the NT5DC family, which shows high sequence similarity with NT5C2. NT5C2 has received attention in the field of hematological neoplasms because NT5C2 mutations have been demonstrated to drive resistance to thiopurine, a drug frequently used to treat hematological neoplasms^35–37^. NT5C2 catalyzes purine-nucleotide metabolism and induces chemotherapeutic resistance via excess export of purines to the extracellular space and depletion of the intracellular purine-nucleotide pool^38–40^. Therefore, NT5DC2 may also participate in purine-nucleotide metabolism and exert purine-nucleotide catalytic activity. A previous study found that NT5DC2 inhibits the tumor sphere formation ability and cell viability of glioblastoma stem cells by downregulating Fyn expression, thus indicating that NT5DC2 also interacts with other proteins and participates in cellular activities other than purine-nucleotide metabolism^21^. Our study reports for the first time that NT5DC2 overexpression promotes HCC cell proliferation and clone formation by regulating the cell cycle and promoting tumor growth in subcutaneous xenografts, while NT5DC2 knockdown inhibits these processes.

Coimmunoprecipitation assays showed that NT5DC2 interacted with 434 cellular proteins, indicating the various functions of NT5DC2 in regulating cellular activity. The HIPPIE database and western blot analysis of the protein expression in NT5DC2-overexpressing and NT5DC2-knockdown cell lines showed that EGFR physically interacted with NT5DC2, and NT5DC2 regulated EGFR expression. Various cellular processes, such as ubiquitination, dephosphorylation and restriction of ligand access, can regulate EGFR levels^41^. Our results revealed that NT5DC2 regulated EGFR expression by downregulating EGFR ubiquitination. Interestingly, both Fyn and EGFR contain protein kinase activity; whether NT5DC2 induced deubiquitination of both proteins in a phosphorylation-deubiquitination-coupled mode requires further verification.

The EGFR signaling pathway has been implicated in HCC pathogenesis^42–44^. EGFR is activated by phosphorylating specific tyrosine kinase residues, and EGFR activation can accelerate intracellular signaling pathways, such as the Src, MAPK and PI3K/Akt pathways^11^. ERK and Akt are thought to be effector molecules downstream of EGFR signaling. Therefore, upregulating EGFR may enhance EGFR downstream signaling and increase ERK and Akt phosphorylation levels. Our results revealed that pERK and Akt phosphorylation levels were enhanced at both S473 and T308 in NT5DC2-overexpressing cells with no changes in total ERK or Akt expression. Erlotinib is a small-molecule EGFR tyrosine kinase inhibitor that inhibits cancer cell growth and metastasis^45,46^. Here, we revealed that NT5DC2 promoted HCC cell proliferation through EGFR-mediated signaling pathways using erlotinib.

NT5DC2 was also overexpressed in HCC tissues via mRNA and protein levels. Moreover, NT5DC2 was related to poor prognoses and early recurrence in HCC patients. Univariate and multivariate analyses showed that NT5DC2 expression was a potential predictor for early recurrence in HCC patients after curative surgery.

In conclusion, the present study demonstrated that NT5DC2 enhanced HCC proliferation by altering the cell cycle and identified a specific interaction between NT5DC2 and EGFR. Furthermore, NT5DC2 regulated EGFR expression by modulating EGFR ubiquitination. These data suggest that NT5DC2 may be a potential predictor and molecular target of tumor recurrence after HCC resection.

## Materials and methods

### Cell cultures

The human HCC cell lines, MHCC-97H, MHCC-97L, MHCC-LM3, Huh7, and PLC/RLF/5, were used in this study. MHCC-97H and PLC/RLF/5 cell line were authenticated by STR analysis and tested for mycoplasma contamination. These cell lines were obtained from the Liver Cancer Institute at Fudan University in Shanghai, China. Cells were cultured in high-glucose Dulbecco’s modified Eagle’s medium (DMEM) supplemented with 10% FBS (Gibco, Gaithersburg, MD, USA) and 1% penicillin/streptomycin under 5% CO_2_ at 37 °C.

### NT5DC2 knockdown and overexpression by transfection

A green fluorescent protein (GFP) tag was designed to the C terminus of NT5DC2 (sequence information from: NM_001134231.1) to express a GFP-NT5DC2 fusion protein. This fusion protein was constructed in the pCDH-CMV-MCS-EF1 vector. The GFP-NT5DC2 lentivirus and vector control were constructed by Yuanmin Biotechnology Co., Ltd. (Shanghai, China). NT5DC2-specific shRNA (GGAGTTTGACCAAGCACATTT) was inserted into the PGMLV-hU6-MCS-CMV-ZsGreen1-PGK-Puro-WPRE vector. The NT5DC2-shRNA lentivirus and vector control were constructed by Genomeditech Co., Ltd. (Shanghai, China). Cells at a density of 3 × 10^5^ were seeded into each well of a six-well plate. The lentiviruses were added to the well with 2 ml of DMEM containing no FBS and 5 μg/ml Polybrene (Sigma, USA). After 24 h, medium containing the virus was removed and replaced with medium containing 10% FBS. The overexpressing and knockdown clones were generated using pooled clone. NT5DC2 expression was then validated via qPCR and western blot.

### RNA isolation and quantitative real-time PCR

RNA isolation and quantitative real-time PCR were performed as previously described^47–50^. Briefly, paired tumor and nontumor specimens were collected from 79 HCC patients after curative resection between 2015 and 2016 at our institute. The Ethics Committee of Zhongshan Hospital at Fudan University approved the use of the tumor tissues, and all patients provided informed consent. Patient follow-up was conducted as previously described. The time interval between resection and tumor recurrence or death was defined as disease-free survival (DFS). Quantitative real-time PCR was performed on RNA extracted from the HCC cell lines or tissues. Total RNA was isolated per the manufacturer’s protocol. The concentration and purity of all RNA samples were determined using the A260–A280 nm ratio. The following primers were used: NT5DC2, 5′-CTTCTCGCTACCGGAGATGG-3′ (forward) and 5′-CCGTCACGTCCTTGTAGAGAT-3′ (reverse); GAPDH, 5′-TGACTTCAACAGCGACACCCA-3′ (forward) and 5′-CACCCTGTTGCTGTAGCCAAA-3′ (reverse); and EGFR, 5’-AGGCACGAGTAACAAGCTCAC-3’ (forward) and 5’-ATGAGGACATAACCAGCCACC-3’(reverse).

### Immunohistochemistry analysis

Immunohistochemistry analysis was performed as previously described^51–56^. The tissue microarray was incubated with rabbit antibody anti-EGFR (1:50; Cell Signaling Technology [CST], USA) overnight. An Ultra-Vision Quanto Detection System HRP DAB (Thermo Fisher Scientific, CA, USA) was used to detect EGFR expression, which was independently evaluated by two pathologists. EGFR expression in the tissue microarray was divided into grades 1–9 for accurate evaluation. Rabbit anti-EGFR antibody (1:50; CST) and rabbit anti-PCNA antibody (1:200, Zuocheng Bio., China) were used for immunohistochemical staining of the subcutaneous xenograft tumors. EGFR expression in the subcutaneous xenograft tumor slides was graded as 0–3 for evaluation. Proliferating cell nuclear antigen (PCNA) expression in the subcutaneous xenograft tumor slides was evaluated from the proportion of PCNA-positive cells.

### Western blot and immunoprecipitation assays

Western blot and immunoprecipitation assays were performed as previously described^57–60^. Total protein was extracted using RIPA lysis buffer with protease inhibitor from HCC cell lines or tissues, then 30 μg of the total protein was subjected to western blot, separated using 12% SDS-PAGE and electrotransferred onto polyvinylidene difluoride membranes (Millipore, Billerica, MA, USA). Membranes were blocked with 5% skim milk, then incubated with the primary antibody. Mouse antibody for GAPDH was purchased from Zsbio (Beijing, China). The rabbit antibodies for NT5DC2 were purchased from Abcam (Cambridge, UK). The rabbit antibodies for EGFR, pAkt308, pAkt473, total Akt, pErk, and total ERK were purchased from CST. The rabbit antibody for the GFP was purchased from Zen-bio (Chengdu, China). The rabbit antibodies for HSPD1 were purchased from Abclonal (Wuhan, China). For immunoprecipitation, GFP-Trap agarose beads were purchased from Chromotek (Germany) and anti-hemagglutinin (HA) magnetic beads were from Bimake (Houston, TX, USA). Cells were lysed using lysis buffer, and the supernatant was collected after centrifugation. Antibody was added to lysates with protein A beads and incubated overnight. The beads were collected and subjected to western blot or in-gel digestion and proteome analysis via liquid chromatography with tandem mass spectrometry (LC-MS/MS). Human Integrated Protein-Protein Interaction rEference (HIPPIE, http://cbdm-01.zdv.uni-mainz.de/~mschaefer/hippie/) was searched for interacting proteins.

### CCK8 assay

CCK8 assays were performed as previously described^51^. Three thousand cells in 200 μl of medium were seeded into a 96-well plate and measured 24, 48, 72, and 96 h after seeding per the manufacturer’s protocol. Cells were incubated in 100 μl of reaction mixture (10 μl CCK-8 and 90 μl DMEM) for 2 h and measured at a wavelength of 450 nm.

### Clone formation assay

Clone formation assays were performed as previously described^51^. One thousand cells were seeded into a six-well plate and cultured under 5% CO_2_ at 37 °C for 2 weeks or 3 weeks. The cells were then fixed with formalin for 30 min and stained with 0.1% crystal violet for 15 min.

### Immunofluorescence assays

Immunofluorescence assays were performed as previously described. In brief, cells were cultured on glass slides with a cell density of 30% in 24-well polystyrene microplates and then fixed with 4% formaldehyde and permeabilized with 0.2% Triton X-100 for 15 min after being washed three times in PBS. After being blocked with 1% BSA, the cells were incubated with primary antibody overnight (4 °C). Cells were washed three times in PBS, then incubated with secondary antibody for 1 h at room temperature. Cells were rinsed with PBS three times, stained with DAPI and analyzed with a confocal laser scanning microscope.

### Flow cytometry

Flow cytometry was performed as previously described^51^. For cell cycle analysis, cells were harvested and fixed in 70% ethanol overnight, stained with propidium iodide and RNase, and analyzed via flow cytometry (BD Biosciences, San Jose, CA, USA). The data were analyzed with Modfit software (Verity Software House, Topsham, ME, USA). For apoptosis analysis, cells were washed with phosphate-buffered saline (PBS) and stained with annexin V and propidium iodide (BD Pharmingen, San Diego, CA, USA). Fluorescence was measured using a FACSCalibur (BD Biosciences, San Jose, CA, USA) and analyzed using FlowJo (Tree Star, Ashland, OR, USA).

### TUNEL staining

Apoptosis of subcutaneous tumor sections were detected by the In Situ Cell Death Detection Kit (Roche, Basel, Switzerland) according to the manufacturer’s instruction. In brief, Sections were incubated in Proteinase K working buffer at 37 degree centigrade for 15 min after deparaffinization and rehydration. Then, they were washed twice with PBS. Sections were immersed with 50 μl TUNEL working solution per sample and incubated at 37 degree centigrade for 30 min in the dark and then washed twice with PBS. Subsequently, sections were immersed with 50 μl converter-POD per sample and incubated at 37 degree centigrade for 30 min in the dark. Then, they were washed twice with PBS and DAB was applied for visualization. Analysis of the staining results was performed as previously described.

### Xenograft models of HCC in nude mice

As described in our previous study, 5-week-old male BALB/c nu/nu mice were purchased from the Shanghai Institute of Materia Medical, Chinese Academy of Science, and housed under specific pathogen-free conditions^61^. The Medical Experimental Animal Care Commission of Zhongshan Hospital approved the experimental protocol. Mice were randomly assigned to the experimental or control group, and cancer cells (5 × 10^6^ cells) in 200 μl of normal saline were implanted via subcutaneous injection to obtain subcutaneous tumors. After three weeks (for the knockdown and control cells) or 5 weeks (for the overexpression and control cells), 5 or 6 mice per group were sacrificed to obtain the tumors.

### Statistical analysis

Statistical analysis was performed using SPSS 19.0 for Windows (SPSS Inc., Chicago, IL, USA). Continuous variables are expressed as the mean ± SD or median (interquartile range). Categorical variables were compared using the *χ*^2^ test or Fisher’s exact test, and continuous variables were compared using Student’s *t*-test or the Mann-Whitney *U*-test. Univariate survival analysis was performed using the Kaplan-Meier (K-M) method, and significant differences between the groups were analyzed using the log-rank test. The relative prognostic significance of the variables for predicting overall survival (OS) and recurrence-free survival (RFS) was assessed using Cox proportional hazards regression models. All statistical tests were two-tailed, and *p* < 0.05 was considered significant. GraphPad Prism 6 (GraphPad Software, San Diego, CA, USA) was also used for statistical analysis.

## Supplementary information

Supplemental Figure legends

Supplemental figure 1

Supplemental figure 2

Supplemental figure 3

Supplemental figure 4

Supplemental figure 5

Supplemental figure 6

Supplemental figure 7

## References

[1] Bray F (2018). Global cancer statistics 2018: GLOBOCAN estimates of incidence and mortality worldwide for 36 cancers in 185 countries. CA Cancer J. Clin..

[2] Global Burden of Disease Cancer, C. et al. Global, regional, and National Cancer Incidence, mortality, years of life lost, years lived With disability, and disability-adjusted life-years for 29 Cancer Groups, 1990 to 2017: a systematic analysis for the global burden of disease study. *JAMA Oncol*. 10.1001/jamaoncol.2019.2996 (2019). 10.1001/jamaoncol.2019.2996 PMC6777271 31560378

[3] Forner A, Reig M, Bruix J (2018). Hepatocellular carcinoma. Lancet.

[4] Villanueva A (2019). Hepatocellular carcinoma. N. Engl. J. Med..

[5] El-Deiry WS (2019). The current state of molecular testing in the treatment of patients with solid tumors, 2019. CA Cancer J. Clin..

[6] Lee YT, Tan YJ, Oon CE (2018). Molecular targeted therapy: treating cancer with specificity. Eur. J. Pharm..

[7] Regorafenib Approved for Liver Cancer. (2017). Cancer Disco..

[8] Mody K, Abou-Alfa GK (2019). Systemic therapy for advanced hepatocellular carcinoma in an evolving landscape. Curr. Treat. Options Oncol..

[9] Al-Salama ZT, Syed YY, Scott LJ (2019). Lenvatinib: a review in hepatocellular carcinoma. Drugs.

[10] Hynes NE, Lane HA (2005). ERBB receptors and cancer: the complexity of targeted inhibitors. Nat. Rev. Cancer.

[11] Yarden Y, Sliwkowski MX (2001). Untangling the ErbB signalling network. Nat. Rev. Mol. Cell Biol..

[12] Bublil EM, Yarden Y (2007). The EGF receptor family: spearheading a merger of signaling and therapeutics. Curr. Opin. Cell Biol..

[13] Schlessinger J (2004). Common and distinct elements in cellular signaling via EGF and FGF receptors. Science.

[14] Gschwind A, Fischer OM, Ullrich A (2004). The discovery of receptor tyrosine kinases: targets for cancer therapy. Nat. Rev. Cancer.

[15] Singh D, Attri BK, Gill RK, Bariwal J (2016). Review on EGFR Inhibitors: critical updates. Mini Rev. Med. Chem..

[16] Zhu AX (2015). SEARCH: a phase III, randomized, double-blind, placebo-controlled trial of sorafenib plus erlotinib in patients with advanced hepatocellular carcinoma. J. Clin. Oncol..

[17] Seifried A, Schultz J, Gohla A (2013). Human HAD phosphatases: structure, mechanism, and roles in health and disease. FEBS J..

[18] Prados J (2015). Borderline personality disorder and childhood maltreatment: a genome-wide methylation analysis. Genes Brain Behav..

[19] van Hulzen KJE (2017). Genetic overlap between attention-deficit/hyperactivity disorder and bipolar disorder: evidence from genome-wide association study meta-analysis. Biol. Psychiatry.

[20] Nakashima A (2019). Identification by nano-LC-MS/MS of NT5DC2 as a protein binding to tyrosine hydroxylase: down-regulation of NT5DC2 by siRNA increases catecholamine synthesis in PC12D cells. Biochem. Biophys. Res. Commun..

[21] Guo S (2019). NT5DC2 promotes tumorigenicity of glioma stem-like cells by upregulating fyn. Cancer Lett..

[22] Budhu A (2006). Prediction of venous metastases, recurrence, and prognosis in hepatocellular carcinoma based on a unique immune response signature of the liver microenvironment. Cancer Cell.

[23] Yuan SX (2016). Long noncoding RNA DANCR increases stemness features of hepatocellular carcinoma by derepression of CTNNB1. Hepatology.

[24] Yuan S (2017). The prediction of clinical outcome in hepatocellular carcinoma based on a six-gene metastasis signature. Clin. Cancer Res..

[25] Menyhart O, Nagy A, Gyorffy B (2018). Determining consistent prognostic biomarkers of overall survival and vascular invasion in hepatocellular carcinoma. R. Soc. Open Sci..

[26] Cancer Genome Atlas Research Network. Electronic address, WBE. & cancer Genome Atlas Research, N. Comprehensive and integrative genomic characterization of hepatocellular carcinoma. *Cell* **169**, 1327–1341. e1323 (2017). 10.1016/j.cell.2017.05.046 PMC5680778 28622513

[27] Chen CH (2006). Decreased expressions of hepsin in human hepatocellular carcinomas. Liver Int.

[28] Ieta K (2007). Identification of overexpressed genes in hepatocellular carcinoma, with special reference to ubiquitin-conjugating enzyme E2C gene expression. Int J. Cancer.

[29] Wei Z (2019). Identification of the potential therapeutic target gene UBE2C in human hepatocellular carcinoma: an investigation based on GEO and TCGA databases. Oncol. Lett..

[30] Wallden K (2007). Crystal structure of human cytosolic 5’-nucleotidase II: insights into allosteric regulation and substrate recognition. J. Biol. Chem..

[31] Ho SR, Lin WC (2018). RNF144A sustains EGFR signaling to promote EGF-dependent cell proliferation. J. Biol. Chem..

[32] Lipkowitz S (2003). The role of the ubiquitination-proteasome pathway in breast cancer: ubiquitin mediated degradation of growth factor receptors in the pathogenesis and treatment of cancer. Breast Cancer Res..

[33] Portolani N (2006). Early and late recurrence after liver resection for hepatocellular carcinoma: prognostic and therapeutic implications. Ann. Surg..

[34] Poon RT (2000). Different risk factors and prognosis for early and late intrahepatic recurrence after resection of hepatocellular carcinoma. Cancer.

[35] Tzoneva G (2018). Clonal evolution mechanisms in NT5C2 mutant-relapsed acute lymphoblastic leukaemia. Nature.

[36] Lu SX, Abdel-Wahab O (2016). Genetic drivers of vulnerability and resistance in relapsed acute lymphoblastic leukemia. Proc. Natl Acad. Sci. USA.

[37] Dieck CL (2018). Structure and mechanisms of NT5C2 mutations driving thiopurine resistance in relapsed lymphoblastic leukemia. Cancer Cell.

[38] Dieck CL, Ferrando A (2019). Genetics and mechanisms of NT5C2-driven chemotherapy resistance in relapsed ALL. Blood.

[39] Moriyama, T. et al. Mechanisms of NT5C2-mediated thiopurine resistance in acute lymphoblastic leukemia. *Mol. Cancer Ther.* **18**, 1887–1895 (2019). 10.1158/1535-7163.MCT-18-1112 PMC6774896 31358663

[40] Tulstrup M (2018). NT5C2 germline variants alter thiopurine metabolism and are associated with acquired NT5C2 relapse mutations in childhood acute lymphoblastic leukaemia. Leukemia.

[41] Citri A, Yarden Y (2006). EGF-ERBB signalling: towards the systems level. Nat. Rev. Mol. Cell Biol..

[42] Ye QH (2016). GOLM1 modulates EGFR/RTK cell-surface recycling to drive hepatocellular carcinoma metastasis. Cancer Cell.

[43] Fuchs BC (2014). Epidermal growth factor receptor inhibition attenuates liver fibrosis and development of hepatocellular carcinoma. Hepatology.

[44] Li J (2014). Monoamine oxidase A suppresses hepatocellular carcinoma metastasis by inhibiting the adrenergic system and its transactivation of EGFR signaling. J. Hepatol..

[45] Baker M (2004). EGFR inhibitors square off at ASCO. Nat. Biotechnol..

[46] Ji H (2006). The impact of human EGFR kinase domain mutations on lung tumorigenesis and in vivo sensitivity to EGFR-targeted therapies. Cancer Cell.

[47] Wang CH (2017). Flot2 promotes tumor growth and metastasis through modulating cell cycle and inducing epithelial-mesenchymal transition of hepatocellular carcinoma. Am. J. Cancer Res..

[48] Xu Y (2017). Sprouty2 correlates with favorable prognosis of gastric adenocarcinoma via suppressing FGFR2-induced ERK phosphorylation and cancer progression. Oncotarget.

[49] Xu YF (2018). Sprouty2 suppresses progression and correlates to favourable prognosis of intrahepatic cholangiocarcinoma via antagonizing FGFR2 signalling. J. Cell Mol. Med..

[50] Liu Z (2019). Transcription factor 7 promotes the progression of perihilar cholangiocarcinoma by inducing the transcription of c-Myc and FOS-like antigen 1. EBioMedicine.

[51] Shi WK (2018). PFKFB3 blockade inhibits hepatocellular carcinoma growth by impairing DNA repair through AKT. Cell Death Dis..

[52] Liu H (2017). Correlations between TBL1XR1 and recurrence of colorectal cancer. Sci. Rep..

[53] Xu YF (2014). Fibroblast growth factor receptor 4 promotes progression and correlates to poor prognosis in cholangiocarcinoma. Biochem. Biophys. Res. Commun..

[54] Xu YF (2015). High-mobility group box 1 expression and lymph node metastasis in intrahepatic cholangiocarcinoma. World J. Gastroenterol..

[55] Xu YF (2019). HMGB1 correlates with angiogenesis and poor prognosis of perihilar cholangiocarcinoma via elevating VEGFR2 of vessel endothelium. Oncogene.

[56] Liu H (2017). Prognostic significance of TBL1XR1 in predicting liver metastasis for early stage colorectal cancer. Surg. Oncol..

[57] Cao MQ (2018). miR-182-5p promotes hepatocellular carcinoma progression by repressing FOXO3a. J. Hematol. Oncol..

[58] Sun R (2019). Annexin10 promotes extrahepatic cholangiocarcinoma metastasis by facilitating EMT via PLA2G4A/PGE2/STAT3 pathway. EBioMedicine.

[59] Wang HM (2014). The catalytic region and PEST domain of PTPN18 distinctly regulate the HER2 phosphorylation and ubiquitination barcodes. Cell Res..

[60] Yang XQ (2014). Clinical significance of nerve growth factor and tropomyosin-receptor-kinase signaling pathway in intrahepatic cholangiocarcinoma. World J. Gastroenterol..

[61] Zhang YY (2018). CD31 regulates metastasis by inducing epithelial-mesenchymal transition in hepatocellular carcinoma via the ITGB1-FAK-Akt signaling pathway. Cancer Lett..

