# Supplementary material for: NT5DC2 promotes tumor cell proliferation by stabilizing EGFR in hepatocellular carcinoma
Source: Cell Death Dis. 2020 May 7;11(5):335. doi: 10.1038/s41419-020-2549-2 (PMC7206140; doi:10.1038/s41419-020-2549-2)
Supplement: Supplementary file 1 — Supplemental Figure legends [file 41419_2020_2549_MOESM1_ESM.docx]

Supplemental Figure 1. Overall survival and disease-free survival plots of twelve genes significantly associated with OS and DFS in Li’s cohort.

Supplemental Figure 2. Correlation between the expression of NT5DC2 and oncogenic markers including DANCR(A), AHCYL2(B), LAMP2(C), SPRY1(D), SERPINA7(E), FGGY(F) and ASLNC16648(G).

Supplemental Figure 3. Expression levels of NT5DC2 on the normal cell line L02 and hepatoma cell lines MHCC97H and PLC/RLF/5.

Supplemental Figure 4. Tumor section slides were analyzed via TUNEL staining for cell apoptosis (A). NT5DC2 overexpression or knockdown showed no effect on the proportions of apoptotic cells (B). ns: no significance.

Supplemental Figure 5. Flow cytometry for detecting cell cycle ratios in MHCC97H-NT5DC2- (A) and PLC/RLF/5-NT5DC2- (B) overexpressing cells compared with their vector controls in starving medium with no FBS for 12 h (left). Statistical analysis of cell cycle phase ratios in MHCC97H-NT5DC2- and PLC/RLF/5-NT5DC2-overexpressing cells (right). *: p < 0.05; **: p < 0.01; ***: p < 0.001.

Supplemental Figure 6. Sequence similarity comparison of NT5DC1, NT5DC2, NT5DC3, NT5DC4 and NT5C2.

Supplemental Figure 7. Effect of EGF stimulation on cell proliferation in control and NT5DC2 overexpressing MHCC97H (A) and PLC/RLF/5 (B) cells.
