# Supplementary figures and images for: NT5DC2 promotes tumor cell proliferation by stabilizing EGFR in hepatocellular carcinoma
Source: Cell Death Dis. 2020 May 7;11(5):335. doi: 10.1038/s41419-020-2549-2 (PMC7206140; doi:10.1038/s41419-020-2549-2)

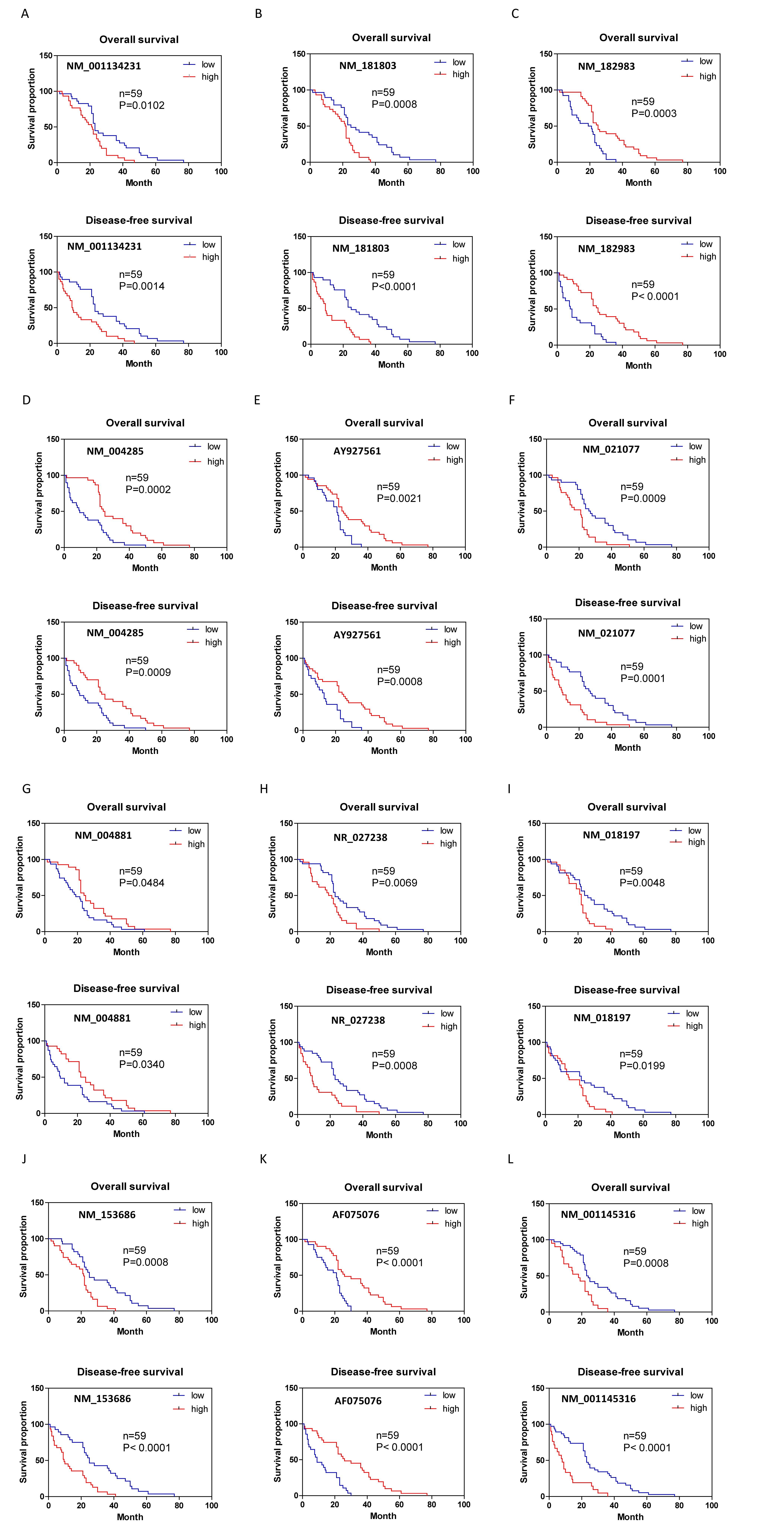

Supplement: Supplementary file 2 — Supplemental figure 1 [file 41419_2020_2549_MOESM2_ESM.tif]

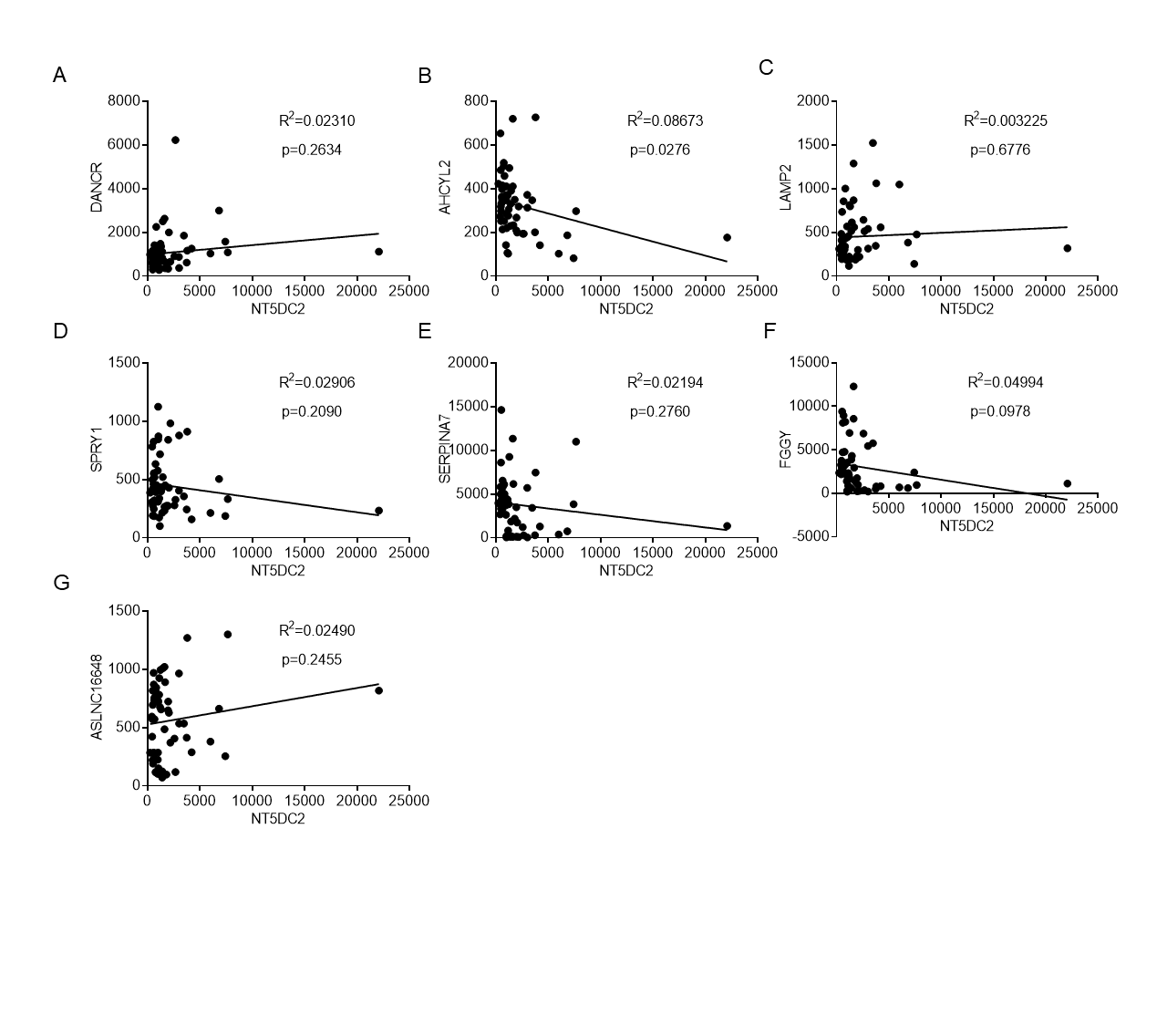

Supplement: Supplementary file 3 — Supplemental figure 2 [file 41419_2020_2549_MOESM3_ESM.tif]

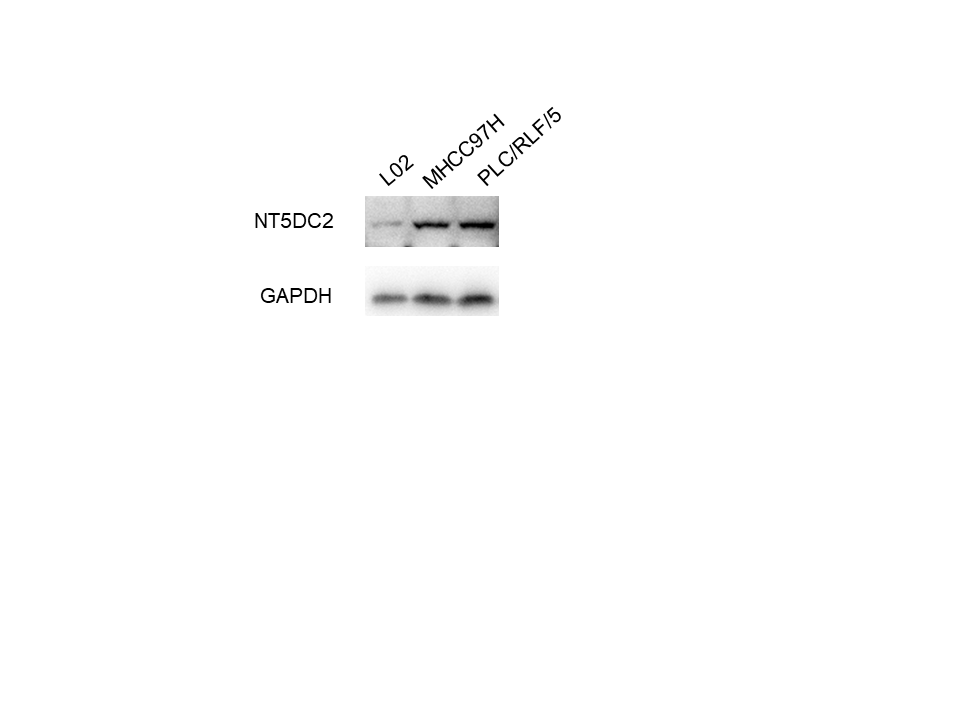

Supplement: Supplementary file 4 — Supplemental figure 3 [file 41419_2020_2549_MOESM4_ESM.tif]

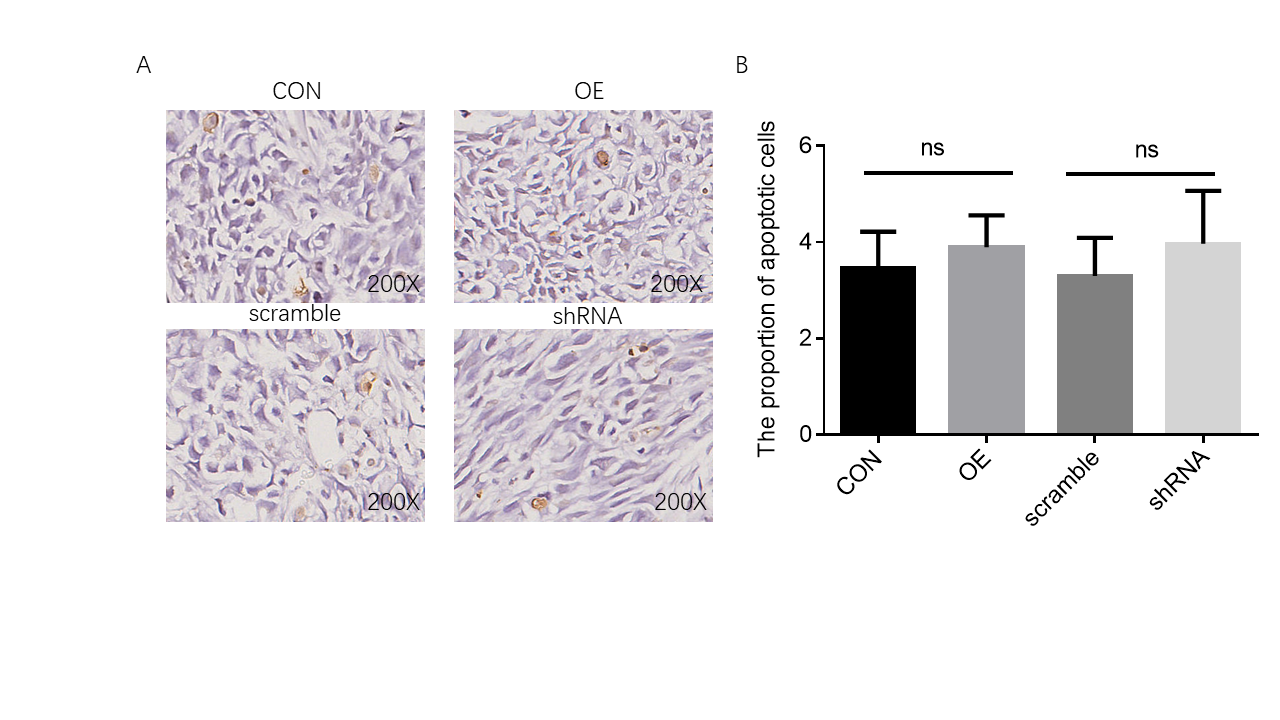

Supplement: Supplementary file 5 — Supplemental figure 4 [file 41419_2020_2549_MOESM5_ESM.tif]

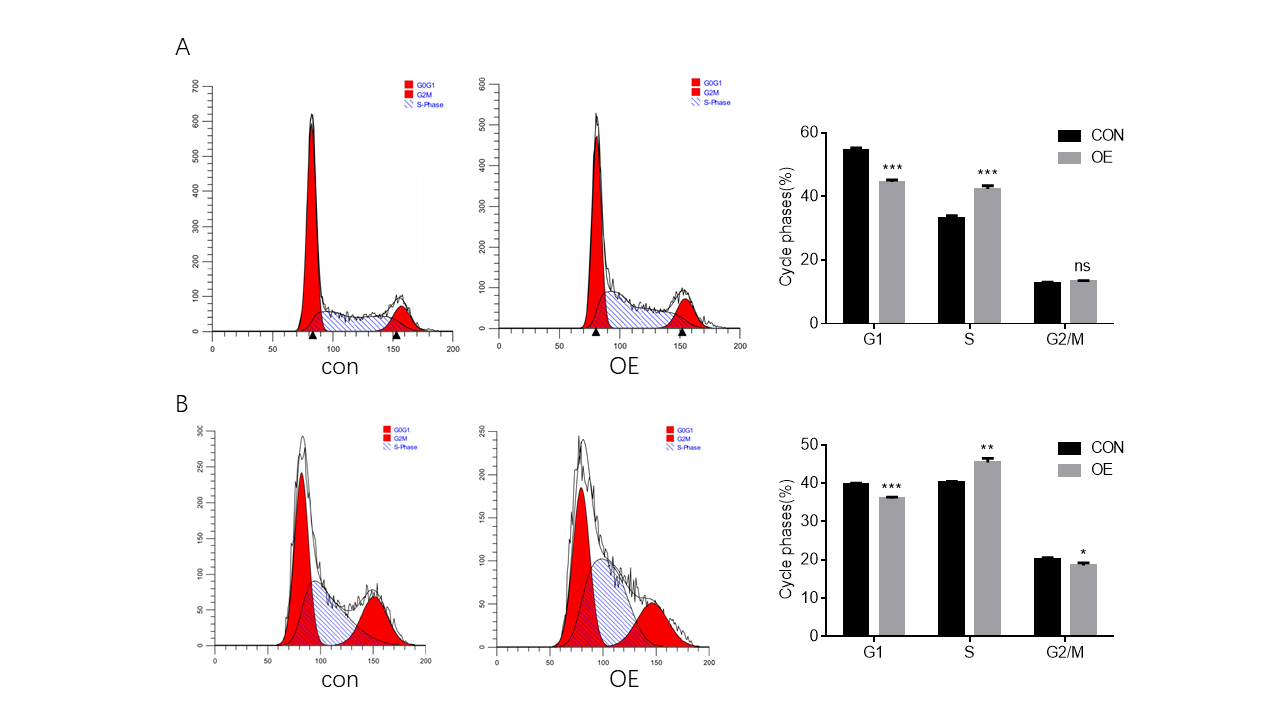

Supplement: Supplementary file 6 — Supplemental figure 5 [file 41419_2020_2549_MOESM6_ESM.tif]

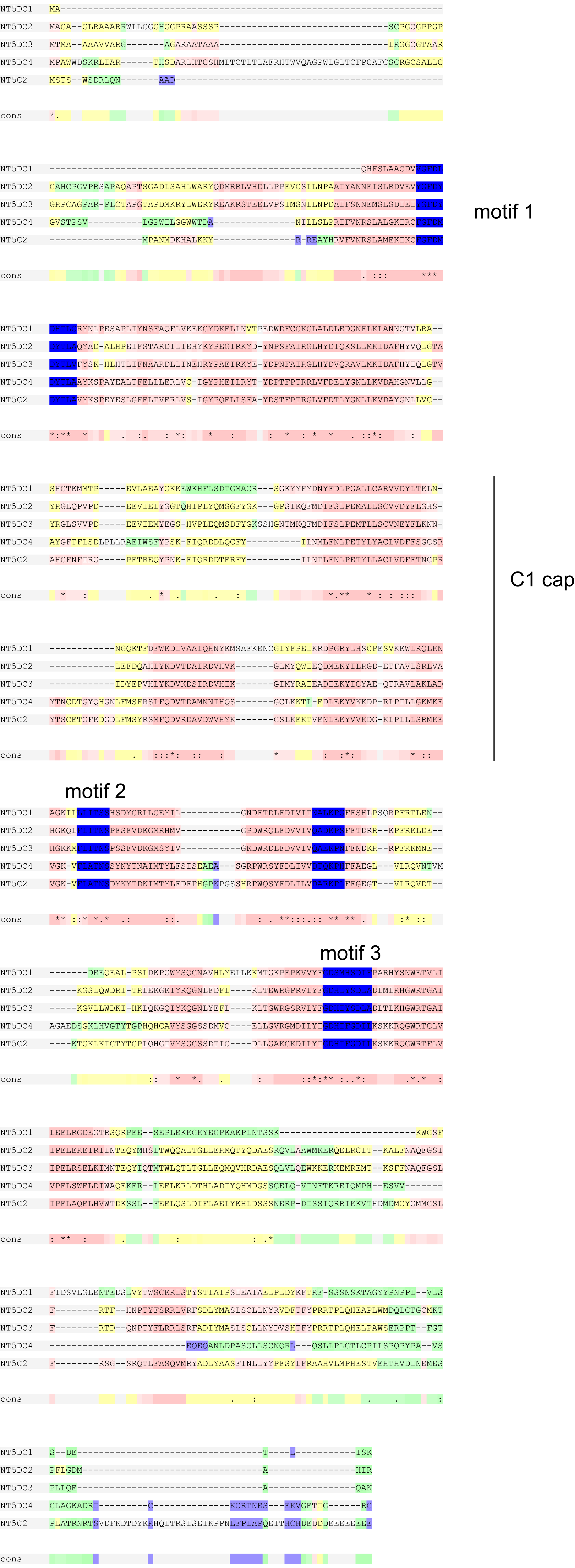

Supplement: Supplementary file 7 — Supplemental figure 6 [file 41419_2020_2549_MOESM7_ESM.tif]

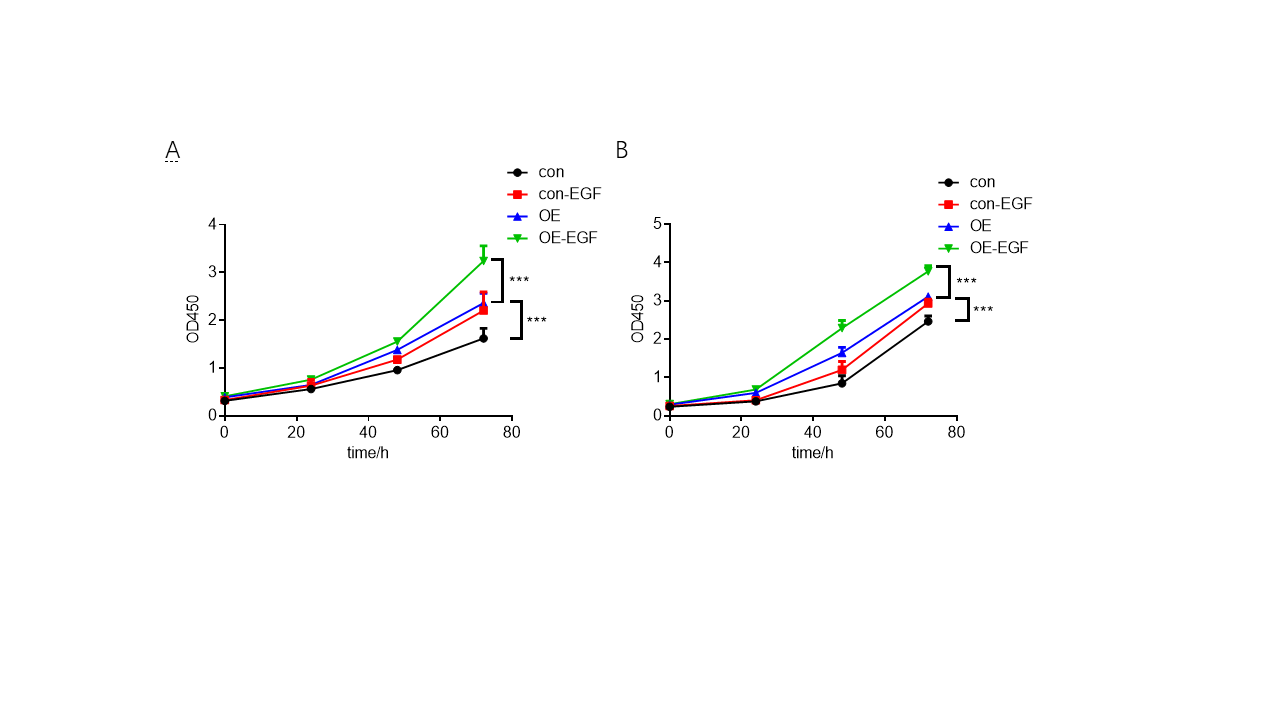

Supplement: Supplementary file 8 — Supplemental figure 7 [file 41419_2020_2549_MOESM8_ESM.tif]
